# YOLO-SDD: An Effective Single-Class Detection Method for Dense Livestock Production

**DOI:** 10.3390/ani15091205

**Published:** 2025-04-23

**Authors:** Yubin Guo, Zhipeng Wu, Baihao You, Lanqi Chen, Jiangsan Zhao, Ximing Li

**Affiliations:** 1 College of Mathematics and Informatics, South China Agricultural University, Guangzhou 510642, China; guoyubin@scau.edu.cn (Y.G.);; 2 Department of Agricultural Technology, Norwegian Institute of Bioeconomy Research (NIBIO), P.O. Box 115, NO-1431 Ås, Norway; 3 Key Laboratory of Smart Agricultural Technology in Tropical South China, Ministry of Agriculture and Rural Affairs, Guangzhou 510642, China

**Keywords:** YOLO, dense object detection, occluded scenarios, attention mechanism, livestock breeding

## Abstract

Single-class object detection, which focuses on identifying, counting, and tracking a specific animal species, plays a vital role in optimizing farm operations. However, dense occlusion among individuals in group activity scenarios remains a major challenge. To address this, we propose YOLO-SDD, a dense detection network designed for single-class densely populated scenarios. First, we introduce a Wavelet-Enhanced Convolution (WEConv) to improve feature extraction under dense occlusion. Following this, we propose an occlusion perception attention mechanism (OPAM), which further enhances the model’s ability to recognize occluded targets by simultaneously leveraging low-level detailed features and high-level semantic features, helping the model better handle occlusion scenarios. Lastly, a Lightweight Shared Head (LS Head) is incorporated and specifically optimized for single-class dense detection tasks, enhancing efficiency while maintaining high detection accuracy. Experimental results on the ChickenFlow dataset, which we developed specifically for broiler detection, show that the n, s, and m variants of YOLO-SDD achieve AP_50:95_ improvements of 2.18%, 2.13%, and 1.62% over YOLOv8n, YOLOv8s, and YOLOv8m, respectively. In addition, our model surpasses the detection performance of the latest real-time detector, YOLOv11. YOLO-SDD also achieves state-of-the-art performance on the publicly available GooseDetect and SheepCounter datasets, confirming its superior detection capability in crowded livestock settings. YOLO-SDD’s high efficiency enables automated livestock tracking and counting in dense conditions, providing a robust solution for precision livestock farming.

## Simple Summary

In dense livestock environments, animals frequently occlude each other, making it challenging for existing object detection methods to accurately identify individual animals. To address this, we propose YOLO-SDD, a single-class dense detection network designed to enhance detection robustness in crowded livestock scenarios. We evaluated it on the ChickenFlow dataset, created specifically for broiler detection, as well as two public datasets for geese and sheep. The results show that YOLO-SDD demonstrates better detection performance compared to popular detectors. This technology provides a reliable automated tool for precision livestock farming, helping to significantly improve the efficiency of farm management.

## 1. Introduction

With the rapid development of modern livestock farming, computer vision technology has become an essential tool for enhancing livestock management, playing a crucial role in animal health management and production monitoring [1,2]. While both single-species and multi-species livestock farming have their respective advantages, single-species systems are often preferred in intensive production settings due to their relatively streamlined management and reduced risk of cross-species disease transmission, particularly in high-density environments [3,4]. In such systems, single-class object detection technology has significant advantages, enabling efficient and accurate identification, counting, and tracking of specific species. As the scale of farming continues to expand, the application of single-class object detection technology is increasingly widespread in single-species farming scenarios, such as poultry and domestic animals [5,6,7]. By integration with automated monitoring technology, it not only improves production efficiency and reduces resource waste but also brings significant economic benefits to the livestock industry. Additionally, this approach effectively reduces stress response in animals during management processes, providing crucial technical support for improving animal welfare.

However, in real-world farming scenarios, particularly in production systems characterized by intensive practices for poultry, the high similarity in farmed animals’ appearance, their dense clustering, and severe occlusion significantly impair the accuracy of detection models [8,9]. Additionally, environmental factors such as variations in lighting, complex backgrounds, and motion blur further exacerbate the challenges of target detection. Early methods, such as Histogram of Oriented Gradients (HOG) [10], relied on hand-crafted features and achieved limited success in simple scenarios, but their feature representation capabilities were constrained under complex backgrounds or occlusion conditions, making them poorly adaptable to diverse scenes and target variations [11].

In recent years, deep learning models such as Regions with Convolutional Neural Network features (R-CNN) [12], Single Shot MultiBox Detector (SSD) [13], and You Only Look Once (YOLO) [14] have seen remarkable progress, driving significant advancements in livestock detection technology. For instance, Tu et al. [15] employed R-CNN for pig detection and segmentation, yielding reliable results in controlled environments, though its computational complexity limits real-time applications. Song et al. [16] applied YOLOv3 to sparsely distributed sheep, achieving a mean Average Precision (mAP) of 97.2%. Yu et al. [17] enhanced YOLOv5 by incorporating a small object detection head to improve individual cow behavior recognition, but the model’s generalization across varying lighting conditions was constrained by training data primarily collected in well-lit environments. Cao et al. [18] developed DenseFCN with point supervision, achieving a 97% counting accuracy for chickens in complex video settings. Lai et al. [19] proposed IO-YOLOv5 for pig detection under dense occlusion and diverse lighting, integrating feature fusion modules to attain a 92.6% mAP. Despite these achievements, convolutional neural networks (CNNs) rely on local convolution operations, struggling to model global contextual information effectively, which reduces target discriminability in dense occlusion and complex backgrounds. Furthermore, during post-processing, detection models are highly sensitive to non-maximum suppression (NMS) thresholds, where setting an appropriate threshold is critical yet challenging. Although improved variants like Soft-NMS [20] enhance overlapping target selection, they still fail to fully address dense scene issues, as valid bounding boxes may be suppressed, impacting overall performance.

To address livestock detection in dense occlusion scenarios, current optimization strategies can be broadly categorized into three types. The first strategy focuses on improving detection precision through loss function optimization. For instance, Hao et al. [21] utilized Focal Loss to dynamically adjust loss weights, significantly reducing missed detections in pig populations. Similarly, Yang et al. [22] employed Repulsion Loss to enhance target separation, effectively improving the detection of deceased chickens. Additionally, Sun et al. [23] introduced SIoU loss to refine bounding box regression accuracy. However, while these improvements excel in specific tasks, they often lack sufficient generalization to handle the diverse complexities of dense occlusion environments. The second category emphasizes enhancing feature representation. For example, the feature fusion technique has been shown to improve chicken detection performance [24], while bilinear feature fusion has proven effective in boosting sheep detection accuracy [25]. While these methods improve multi-scale object detection, they may not capture the fine-grained local features necessary for accurately distinguishing objects in dense occlusion environments. The third category employs attention mechanisms to emphasize critical regions, with methods like channel attention [26,27], spatial attention [28], and Convolutional Block Attention Module (CBAM) [29,30] being widely applied in livestock detection. However, these mechanisms may easily result in a significant increase in the number of parameters and computational load. Additionally, they fail to fully leverage the relationship between low-level detailed features and high-level semantic features, which is crucial for resolving occlusion in densely packed scenarios.

While object detection in dense environments has experienced some progress, significant challenges still exist, especially when dealing with frequent occlusions where limited features can be extracted. Additionally, existing methods often improve performance by simply superimposing more complex modules and consequently increasing computational complexity and the number of parameters. To address the aforementioned challenges, we propose YOLO-SDD, a network designed for single-class dense detection. First, we introduce Wavelet-Enhanced Convolution (WEConv) to capture spatial information across various scales and frequencies, thereby improving the model’s feature extraction ability for dense and edge targets. Next, to further enhance the model’s robustness in occluded environments, we design the Occlusion Perceptual Attention Module (OPAM), which effectively integrates low-level detailed features with high-level semantic features to improve the model’s discriminative ability. Furthermore, we propose Lightweight Shared Head (LS Head), which not only allows the detection head to learn common spatial features across multiple detection layers through a shared convolutional structure but also significantly reduces computational overhead and model parameters. The main contributions of this paper are listed as follows:

1. We propose a single-class object detection framework called YOLO-SDD, specifically designed for the detection of poultry (e.g., chickens and geese) and sheep. To enhance the model’s detection performance and efficiency in complex occlusion scenarios, we introduce WEConv, LS Head, and OPAM.
2. We establish a dataset named ChickenFlow, focusing on scenarios with dense occlusion among flocks of chickens. This dataset enriches existing resources for object detection and provides crucial data support for research in occluded object detection in dense environments.
3. Extensive experiments are conducted on three livestock farming datasets involving chickens, geese, and sheep, comparing YOLO-SDD with seven of the most popular object detectors. The results demonstrate that YOLO-SDD excels in both accuracy and robustness. Additionally, ablation studies validate the contributions of each module to the improved performance and efficiency in detecting objects.

## 2. Materials and Methods

### 2.1. Dataset

In this study, we use three animal detection datasets, including the ChickenFlow dataset, a dataset specifically created for this research, and two public datasets: GooseDetect [31] and SheepCounter [32]. These datasets cover diverse agricultural scenarios, allowing for an in-depth assessment of model capabilities in handling occlusion, object density, and scale variations.

#### 2.1.1. ChickenFlow

**Figure 1:**
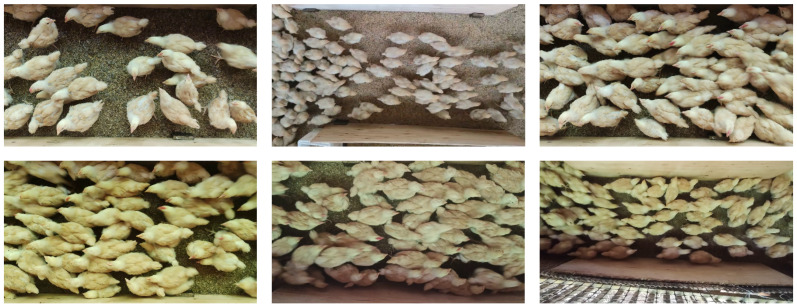
Typical images from the ChickenFlow, characterized by high density and occlusion.

The experimental data in this study were collected from a small-scale broiler farm that adopted a floor-based system, with approximately 400–500 broiler chickens. The data were captured in a corridor area measuring 1.2 meter × 0.5 meter, reflecting the movement of broilers under varying conditions. A Redmi K30 Pro smartphone, equipped with a 64 million pixels main camera, was positioned 1 m above the corridor floor to record videos at a resolution of 1920 × 1080 pixels and 30 frames per second. The key frames were extracted and converted to high-quality PNG images. Video collection was mainly conducted from noon to afternoon under natural lighting conditions. Due to variations in time and shooting angles, there was a weaker illumination of some broilers in certain images. In some scenes, the rapid movement of broilers often caused localized blurring. In scenes with more broilers, crowding and occlusion frequently occurred, with some targets typically appearing near the edges. Typical collected images are shown in Figure 1.

**Figure 2:**
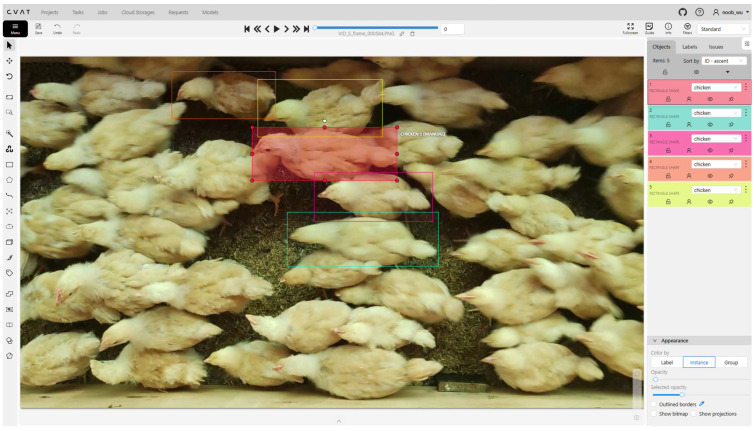
Image annotation process. Different colors represent different annotated instances.

After collecting the broiler images, a selection process was conducted to remove low-quality images that were excessively blurred or overexposed, prioritizing clear and well-lit photos. To further enhance annotation accuracy, the open-source annotation tool CVAT (v2.5.1, Intel Corporation, Santa Clara, CA, USA) [33] was used for labeling. Each broiler in the images was precisely marked to ensure clear and accurate bounding boxes, as illustrated in Figure 2. The annotation process not only included outlining the broilers’ shapes but also assigning corresponding category labels to facilitate subsequent object detection tasks. Ultimately, the ChickenFlow dataset consists of 4718 images with 90,717 annotated instances. The dataset was divided into training, validation, and test sets in a 7:1:2 ratio, comprising 3309, 464, and 945 images, respectively. To quantify the degree of occlusion among objects in complex scenes, we define the Overlap Ratio (OR), which measures the spatial overlap interference between bounding boxes. The computation is shown in Equation (1).

(1) $$\mathrm{OR}\left(B_{i}\right)=\frac{\mathrm{Area}\left({⋃}_{j\neq i}\left(B_{i}\cap B_{j}\right)\right)}{\mathrm{Area}(B_{i})}$$

where $B_{i}$ denotes the bounding box of the *i*-th object and $B_{j}$ represents the bounding boxes of all other objects in the image (j≠i). The numerator calculates the total area of overlap between $B_{i}$ and all other $B_{j}$, with the union operator ensuring that overlapping regions are not double-counted. The denominator corresponds to the area of $B_{i}$ itself. A higher OR value indicates a greater degree of occlusion, quantifying the extent to which $B_{i}$ is interfered with by neighboring objects.

For each image, we define its OR as the average OR of all annotated objects within the image. The average OR in the ChickenFlow is 0.29, with 746 images exhibiting an OR greater than 0.5. To further evaluate the performance of detection models in high-density and heavily occluded scenarios, we selected a subset of 143 images from the test set that meet two conditions: (1) the OR of each image exceeds 0.5 and (2) the number of objects in the image is greater than 40. This subset provides a challenging yet representative benchmark for assessing detection performance under severe occlusion and crowding.

#### 2.1.2. GooseDetect and SheepCounter

**Figure 3:**
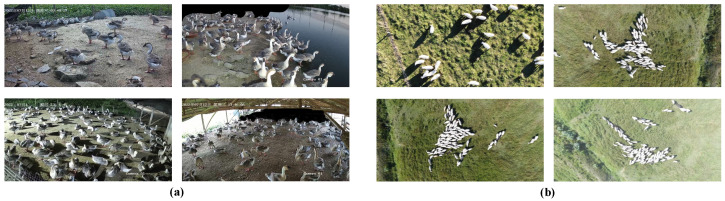
Representative images from the GooseDetect and SheepCounter datasets. (**a**) Representative images from GooseDetect, featuring significant occlusion and complex backgrounds. (**b**) Images from the SheepCounter dataset, showing moderate target density with mild occlusion, but a large number of small targets.

GooseDetect. The GooseDetect dataset was collected from a goose farm and consists of 2147 training images, 256 validation images, and 257 test images, with a total of 98,111 annotated instances. This dataset encompasses diverse scenarios, including fenced enclosures as well as free-range areas without barriers. Geese in the images are often occluded by environmental obstacles, such as fences and water dispensers, as well as by other geese, resulting in significant occlusion challenges. Moreover, variations in camera perspectives, including low-angle shots from the ground and slightly elevated viewpoints, further enhance the diversity of the dataset, as illustrated in Figure 3a.

SheepCounter. The SheepCounter dataset is captured from a high altitude using Unmanned Aerial Vehicles and includes 1203 training images, 350 validation images, and 174 test images, with a total of 55,435 annotated targets. Although occlusion in this dataset is relatively mild, there are still many small and densely packed sheep. Meanwhile, the diversity of shooting angles, along with variations in lighting conditions and weather, also affect the quality of the images. Consequently, these factors pose challenges to the precision of the object detection model. An example of typical SheepCounter images is shown in Figure 3b.

### 2.2. YOLO-SDD Model Construction

#### 2.2.1. Overall Architecture

**Figure 4:**
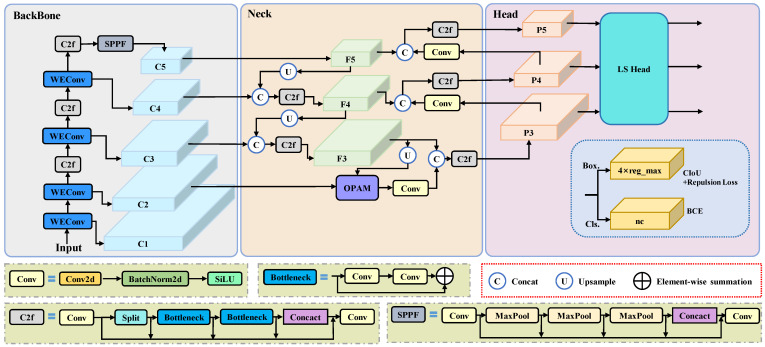
Overview of the network architecture of YOLO-SDD.

We select the YOLOv8 [34] object detector as our baseline model. As shown in Figure 4, we break down the architecture of YOLO-SDD into three main components: Backbone, Neck, and Head. The backbone of YOLO-SDD consists of a series of convolutional layers, with WEConv for more effective feature extraction at different scales and frequencies of dense and edge targets. The C2f module further strengthens feature fusion capability through a cross-layer connection mechanism. At the beginning, the input feature maps are divided into two parts: one part is passed directly, while the other part undergoes feature extraction through multiple Bottleneck modules. The results are then concatenated along the channel dimension to achieve cross-channel feature fusion. The SPPF module performs multi-scale pooling to compress the feature maps to a fixed size, effectively retaining global context information while reducing computational complexity. After processing by the backbone, the model generates a series of feature layers: C1, C2, C3, C4, and C5. In the neck, the OPAM is introduced to fuse low-level features from C2 with high-level features from F3, enhancing the model’s feature representation. This fusion improves the model’s ability to accurately detect and distinguish targets in densely occluded environments. Finally, in the head, the model generates diversified output at three different resolutions. The LS Head employs a shared convolutional design for predicting object bounding boxes and classes, reducing the number of parameters while improving detection accuracy for single-class dense targets. The loss for bounding box prediction is computed using CIoU loss and repulsion loss [35], while binary cross-entropy is used as the classification loss. The key hyperparameters of the YOLO-SDD model can be found in Table A1.

#### 2.2.2. Wavelet-Enhanced Convolution

**Figure 5:**
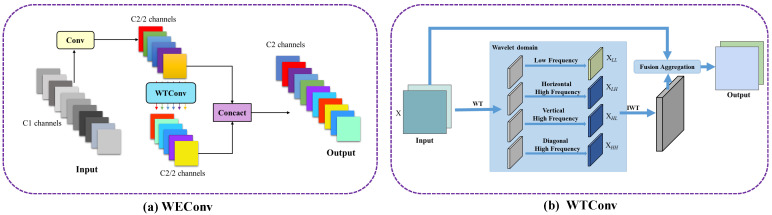
The structure of the WEConv, which utilizes WTConv as a low-cost operation module.

To reduce the high computational cost and large number of parameters typically associated with traditional convolution operations, GhostNet [36] generates more feature maps with fewer parameters, making it an efficient choice for feature extraction. However, Depthwise Convolution (DWConv) [37], the low-cost operation module of GhostNet, still suffers from certain limitations. DWConv uses fixed-size convolution kernels (such as 3 × 3 and 5 × 5), which limits its ability to capture multi-scale features and thus constrains its spatial information extraction capacity. Moreover, DWConv operates solely in the spatial domain, neglecting the processing of frequency domain features, which prevents it from fully exploiting the multi-scale and frequency information of input features. To overcome these issues and further improve the model’s performance in target detection under dense occlusion and complex environments, we propose WEConv, as shown in Figure 5a. By incorporating wavelet transform [38] into the convolution operation, WEConv significantly enhances the ability of the convolution layer to capture local details.

The WEConv first applies a 1 × 1 convolution to the input feature map *X* to generate a set of feature maps, which are then downsampled to half the size of the original input. These feature maps are then passed to the Wavelet Transform Convolution (WTConv), which decomposes the spatial frequency components of the feature maps using Haar wavelet transform. As shown in Figure 5b, WTConv uses four filters to decompose the input features, denoted as $X_{LL}$, $X_{LH}$, $X_{HL}$, and $X_{HH}$. Here, $X_{LL}$ represents the low-frequency component of the input feature map, while $X_{LH}$, $X_{HL}$, and $X_{HH}$ correspond to the high-frequency components of the input feature map in the horizontal, vertical, and diagonal directions, respectively. After decomposition, these processed components are recombined using the inverse wavelet transform (IWT) to generate the feature map. Finally, this map is fused with the input features to output the enhanced feature map. This process allows WEConv to capture spatial information across multiple scales and frequencies, effectively compensating for the information loss that may occur in depthwise convolution. Additionally, wavelet decomposition helps to retain edge information, which is crucial for detecting fine-grained details and target boundaries, especially in dense or occluded scenes.

#### 2.2.3. Occlusion Perceptual Attention Module

**Figure 6:**
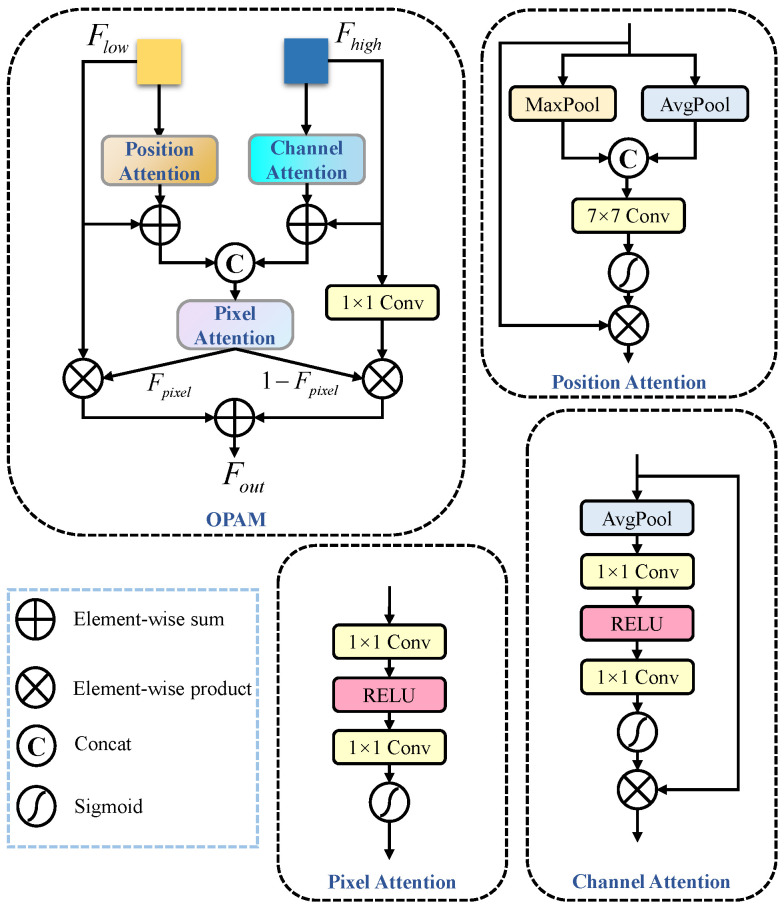
Occlusion Perceptual Attention Module.

The unique low-level features of the C2 layer, which contain rich detail and spatial positional information, provide distinct advantages in detecting small targets and occlusions. However, directly utilizing the C2 layer features by adding an additional xsmall head [39] can enhance the model’s ability to handle occluded scenes, but it significantly increases computational complexity and fails to fully exploit the potential spatial information of the C2 layer. Therefore, we propose the OPAM, as shown in Figure 6, which enhances feature representation without adding a new detection head.

OPAM introduces a multidimensional attention mechanism to adaptively enhance key regions of the input features, improving the precision of feature representation by focusing on the most important areas for detection. It first encodes low-level and high-level features through a feature extraction network. The Position Attention receives input from low-level features, which typically contain rich spatial positional information and details. It enhances the spatial representation by capturing spatial relationships between targets, especially the subtle changes in occluded scenarios. On the other hand, high-level features are input into the Channel Attention, which assigns adaptive weights to different channels, enhancing channels that contain key semantic information while weakening channels that carry redundant information. However, directly summing the features processed by the Position Attention and Channel Attention modules may result in semantic mismatches. To address this, we input the low-level features along with their corresponding high-level features into the Pixel Attention to obtain adaptive weights.

In OPAM, the Positional Attention takes the low-level feature map $F_{low}$ from the C2 layer as input, and generates the positional attention feature map $F_{pa}$. Meanwhile, the Channel Attention processes the high-level feature map $F_{high}$ from the F3 layer to produce the channel attention feature map $F_{ca}$. Subsequently, the Pixel Attention receives two inputs: $F_{low}+F_{pa}$ and $F_{high}+F_{ca}$. The Pixel Attention generates pixel-level attention weights $F_{pixel}$, dynamically computed using a Sigmoid activation function to map attention scores to the range [0,1]. Finally, the adjusted feature maps are refused, as shown in Equation (2).

(2) $$F_{out}=F_{low}⊙F_{pixel}+Conv_{1\times 1}\left(F_{high}\right)⊙\left(1-F_{pixel}\right)$$

where $F_{low}$ is element-wise multiplied by $F_{pixel}$, which applies a weighting to the low-level features based on the attention map, enhancing the representation of low-level features in the focused regions. Then, $F_{high}$ is passed through a 1×1 convolution and element-wise multiplied by $1-F_{pixel}$, applying a weight to the high-level features. This causes the model to rely more on high-level features in regions where the attention map indicates less focus. Finally, the two weighted feature maps are summed to generate the final output feature map $F_{out}$. This approach allows the model to flexibly combine low-level and high-level features according to the attention map, enhancing feature representation.

#### 2.2.4. Lightweight Shared Head

In single-class detection scenarios, we observe a correlation between class information and the position of the bounding box. Additionally, inspired by FCOS [40], which shows that Group Normalization (GN) can enhance the performance of detection heads, this work adopts a similar approach. To address the inconsistencies that emerge when handling objects of varying scales, we propose Group Normalization Convolution (GNConv) as a shared convolutional structure. GNConv aims to normalize feature distributions, thereby alleviating scale-related feature inconsistencies.

**Figure 7:**
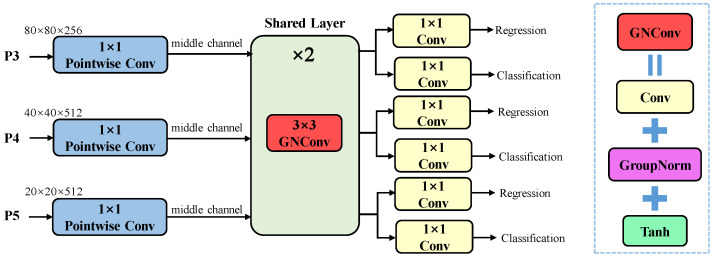
Lightweight Shared Head.

As shown in Figure 7, we propose the LS Head, which reduces the redundancy caused by designing separate convolutional layers for each task in the decoupled head by sharing convolutional layers among multiple detection layers. Specifically, the P3, P4, and P5 feature maps are first processed through three 1 × 1 pointwise convolutions for feature extraction and dimensionality reduction, ensuring that the resulting middle channel dimensions remain consistent across all feature levels. These are then passed through shared layers composed of two 3 × 3 GNConv blocks, enabling the sharing of spatial information across different feature layers. Finally, the feature maps are passed through classification and regression branches to output class and bounding box information. This shared structure effectively reduces the model’s parameters and overall size, making it more lightweight. Additionally, the simplified architecture decreases the complexity of hyperparameter tuning, accelerating model convergence. In single-class detection scenarios, the shared head effectively learns the spatial relationships between classes and bounding boxes, achieving detection performance comparable to or even exceeding that of the decoupled head while using fewer parameters.

#### 2.2.5. Occlusion Loss

Occlusion often leads to a decline in the regression accuracy of bounding boxes. To address this challenge, we introduce the repulsion loss, which consists of two components: RepGT and RepBox. Therefore, our occlusion loss can be defined as a combination of the following three parts:

(3) $$L=L_{CIoU}+\alpha \times L_{RepGT}+\beta \times L_{RepBox}$$

where $L_{CIoU}$ serves as the attraction term, compelling the predicted bounding box to approach its corresponding ground truth box, where CIoU is utilized. $L_{RepGT}$ and $L_{RepBox}$ act as repulsion terms. Coefficients α and β are used to balance the weights of the auxiliary losses. The definition of $L_{CIoU}$ is as follows:

(4) $$L_{CIoU}=1-\mathrm{IoU}+\frac{\rho^{2}\left(b,b^{gt}\right)}{c^{2}}+\omega \nu ,\omega =\frac{v}{(1-\mathrm{IoU})+v},v=\frac{4}{\pi^{2}}{\left(\arctan\frac{w^{gt}}{h^{gt}}-\arctan\frac{w}{h}\right)}^{2}$$

where IoU represents the Intersection over Union between the predicted box and the ground truth box. $\rho^{2}\left(b,b^{gt}\right)$ denotes the Euclidean distance between the center points of the predicted box *b* and the ground truth box $b^{gt}$. *c* refers to the length of the diagonal of the smallest enclosing rectangle that contains both the predicted and ground truth boxes. ω is a weight term that measures the consistency of aspect ratios, while *v* indicates the difference in aspect ratios between the predicted box and the ground truth box.

The purpose of RepGT is to encourage the current bounding box to be as far away as possible from the surrounding ground truth boxes, thereby reducing overlap and improving localization accuracy. The definition of $L_{\mathrm{RepGT}}$ is as follows:

(5) $$L_{\mathrm{RepGT}}=\frac{\sum_{P\in P^{+}}{\mathrm{Smooth}}_{\ln}\left(\mathrm{IoG}\left(P,G_{Rep}^{P}\right)\right)}{|P^{+}|},{\mathrm{Smooth}}_{ln}=\left\{\begin{array}{cc} -\ln(1-x) & x\leq \sigma \\ \frac{x-\sigma }{1-\sigma }-\ln\left(1-\sigma \right) & x>\sigma \end{array}\right.$$

where $P\in P^{+}$ represents the set of all positive samples, and $G_{Rep}^{P}$ denotes the ground truth with the maximum IoU surrounding the target. The overlap between *P* and $G_{Rep}^{P}$ is defined as the intersection of ground truth (IoG): $\mathrm{IoG}\left(P,G\right)=\frac{\mathrm{area}(P\cap G)}{\mathrm{area}(G)}$ and IoG(P,G)∈[0,1]. ${\mathrm{Smooth}}_{ln}$ is a smooth and continuous differentiable logarithmic function defined in the interval (0,1), which is used to adjust the sensitivity of the repulsion loss to outliers.

The purpose of the RepBox is to push the predicted boxes as far away as possible from the surrounding predicted boxes and to reduce the IoU between them. The equation for $L_{RepBox}$ is as follows:

(6) $$L_{RepBox}=\frac{\sum_{i\neq j}{\mathrm{Smooth}}_{\ln}\left(\mathrm{IoU}\left(B^{p_{i}},B^{p_{j}}\right)\right)}{\sum_{i\neq j}1\left[\mathrm{IoU}\left(B^{p_{i}},B^{p_{j}}\right)>0\right]+\epsilon }$$

where 1 is the identity function and ϵ is a very small constant to avoid division by zero. For the predicted bounding boxes $B^{p_{i}}$ and $B^{p_{j}}$ between different groups $P_{i}$ and $P_{j}$, we aim to minimize the overlapping area between them.

### 2.3. Evaluation Metrics

We use AP as the primary evaluation metric. The AP for each class is calculated based on precision and recall, which are defined as follows:

(7) $$\mathrm{Precision}=\frac{TP}{TP+FP}$$

(8) $$\mathrm{Recall}=\frac{TP}{TP+FN}$$

where TP, FP, and FN represent true positives, false positives, and false negatives, respectively. The definitions of positive and negative samples depend on the IoU threshold. For example, AP_50:95_ is calculated using IoU thresholds ranging from 0.5 to 0.95 with a step size of 0.05, while ${\mathrm{AP}}_{50}$ is computed with an IoU threshold of 0.5, and ${\mathrm{AP}}_{75}$ with an IoU threshold of 0.75. By varying the confidence threshold, different combinations of precision and recall values are obtained, which together form a precision–recall (P-R) curve. The AP for each class is then computed as the area under the corresponding P-R curve, and the mean AP is averaged across all classes.

### 2.4. Experimental Setup

We utilized a server equipped with an NVIDIA GeForce RTX 4090 GPU to ensure efficient computation and training processes. The training environment was set up with CUDA version 12.1 and Python 3.8.0. For the training process, we employed the AdamW optimizer, with an initial learning rate set to $2\times 10^{-3}$ and a weight decay of $5\times 10^{-4}$. The training included a warm-up phase of 3 epochs with an initial momentum of 0.9. To ensure fairness and comparability of the model performance, no pre-trained weights were used in the training process of all comparative experiments and ablation experiments. A batch size of 16 was used throughout the training, and a total of 200 epochs were trained. The weight parameters α and β in the occlusion loss were set to 0.4 and 0.6, respectively. Additionally, mosaic augmentation was disabled for the final 10 epochs. Finally, we evaluated three variants of the YOLO-SDD model: YOLO-SDD-n (a lightweight version), YOLO-SDD-s (a small-scale version), and YOLO-SDD-m (a medium-scale version). These variants differ in model size and complexity, with YOLO-SDD-n designed for resource-constrained environments, YOLO-SDD-s offering a balanced trade-off between size and performance, and YOLO-SDD-m providing higher average precision and performance on more powerful computational devices.

## 3. Results and Discussion

### 3.1. Comparison with State-of-the-Arts

In order to assess the performance of the proposed model, a comparative experiment was conducted with a series of object detection algorithms, including YOLOv5 [41], YOLOv7 [42], YOLOv8 [34], YOLOv9 [43], YOLOv10 [44], YOLOv11 [45], and Mamba-YOLO [46].

**Figure 8:**
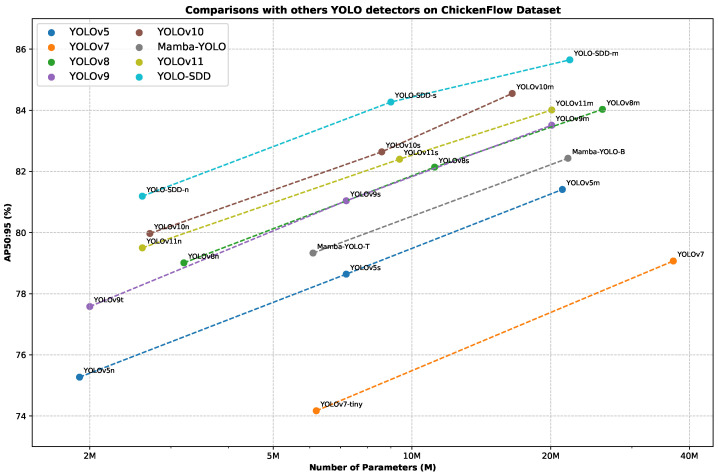
Comparisons with others YOLO detectors on ChickenFlow dataset.

In Table 1, we present a comprehensive comparison of our proposed YOLO-SDD with other leading real-time object detection models on the ChickenFlow dataset. First, we compare YOLO-SDD with our baseline model, YOLOv8. Our YOLO-SDD achieves an improvement of 2.18%, 2.13%, and 1.62% in AP_50:95_, while reducing parameters by 18.75%, 19.64%, and 15.06% compared to the N, S, and M variants, respectively. Additionally, YOLO-SDD reduces FLOPs by 1.8 G in relation to the variant M. This demonstrates the effectiveness of our model design, particularly the incorporation of the OPAM and WEConv in enhancing the model’s robustness in dense occlusion scenarios. Compared to other popular YOLO detectors, our proposed YOLO-SDD shows significant improvements across all scales. Specifically, YOLO-SDD-n outperforms the best-performing lightweight model, YOLOv10n, by 1.22% in AP_50:95_, with only a minor increase of 0.4 M parameters. For small-scale models, the YOLO-SDD-s achieves an increase of 1.63% and 1.87% in AP_50:95_ over the YOLOv10s and YOLOv11s, respectively, while maintaining a competitive parameter size. On the medium scale, YOLO-SDD-m outperforms YOLOv10m by 1.1% in AP_50:95_, achieving state-of-the-art performance among medium-sized YOLO variants. Furthermore, compared to YOLOv7, YOLO-SDD-m reduces the number of parameters and FLOPs by 14.9 M and 27.6 G, respectively, while improving AP_50:95_ by 6.58%. The reduction in parameter count is largely attributed to the design of the Lightweight Head (LS head), which optimizes feature processing while maintaining high detection accuracy. To provide a more intuitive comparison of the parameter size and accuracy of various detection models on the ChickenFlow dataset, we present a comparative analysis of different object detection methods. As shown in Figure 8, YOLO-SDD achieves the best balance between parameter size and accuracy. These experimental results validate the effectiveness of the YOLO-SDD architecture and demonstrate that the model consistently exhibits outstanding performance across different variants.

Additionally, as shown in Table 2, we compare the performance of various state-of-the-art object detection models in dense occlusion scenes of the ChickenFlow dataset. Our proposed YOLO-SDD-s achieves the highest detection metrics across all evaluation criteria, with AP_50:95_ of 75.60%, ${\mathrm{AP}}_{50}$ of 94.04%, and ${\mathrm{AP}}_{75}$ of 85.38%. Compared to the strong baseline YOLOv8s, YOLO-SDD-s shows improvements of 3.13% in AP_50:95_, 0.87% in ${\mathrm{AP}}_{50}$, and 1.61% in ${\mathrm{AP}}_{75}$. Compared to the second-best-performing YOLOv10s, YOLO-SDD-s achieves a margin of 0.51% under the less strict ${\mathrm{AP}}_{50}$ metric and a more notable improvement of 1.41% under the stricter ${\mathrm{AP}}_{75}$ metric. Furthermore, compared to the latest detector YOLOv11s, YOLO-SDD-s achieves gains of 3.06% in ${\mathrm{AP}}_{50:95}$ and 2.81% in ${\mathrm{AP}}_{75}$. These results demonstrate the effectiveness of YOLO-SDD-s in handling severe occlusion and densely crowded scenarios.

**Figure 9:**
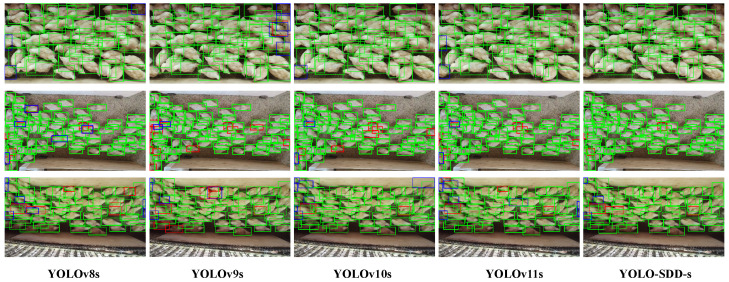
Detection results of YOLOv8s, YOLOv9s, YOLOv10s, YOLOv11s, and YOLO-SDD-s with an IoU threshold of 0.65. Green boxes indicate correctly detected objects, red boxes represent missed detections, and blue boxes denote false positives.

To visually compare the detection performance of YOLO-SDD with other detectors, Figure 9 presents representative detection result visualizations from the ChickenFlow dataset. In the first row, which depicts a medium-density scene with minimal occlusion between broilers, YOLO-SDD-s successfully identifies all targets. In contrast, YOLOv8s, YOLOv10s, and YOLOv11s exhibit fewer false positives (blue boxes), while YOLOv9s performs the worst, showing both false positives and misses (red boxes). In the second row, captured from a greater distance, other detectors display a higher rate of false positives and misses. However, YOLO-SDD-s stands out with the fewest errors, demonstrating superior performance. In scenes with dense distribution and partial occlusion, YOLOv8s tends to misidentify overlapping broilers as multiple separate targets, whereas YOLO-SDD-s more accurately delineates their bounding boxes. In the third row, where varying illumination and motion blur affect the broiler targets, all detectors experience some degree of misses and false positives. Nevertheless, YOLO-SDD-s maintains the best detection performance. YOLOv10s also achieves relatively good results but occasionally misclassifies wooden boards—similar in color to broilers—as targets. YOLOv9s, however, shows the weakest performance, with a higher number of erroneous detections. These experiments demonstrate that YOLO-SDD-s captures targets more accurately in dense occlusion scenarios, offering more reliable detection performance compared to existing models.

### 3.2. Experiments on GooseDetect and SheepCounter

The experimental results on the GooseDetect are shown in Table 3. YOLO-SDD-s outperforms all other methods across all metrics on this dataset. Specifically, YOLO-SDD-s achieves an AP_50:95_ of 56.47%, an ${\mathrm{AP}}_{50}$ of 91.71%, and an ${\mathrm{AP}}_{75}$ of 60.68%. Compared to the baseline method YOLOv8s, YOLO-SDD-s improves AP_50:95_, ${\mathrm{AP}}_{50}$, and ${\mathrm{AP}}_{75}$ by 1.62%, 1.26%, and 2.67%, respectively. In the more demanding ${\mathrm{AP}}_{75}$ metric, YOLO-SDD-s surpasses the closest-performing YOLOv9s by 0.32 percentage points and outperforms the classic YOLOv5s by 4.71%. Furthermore, compared to the latest YOLOv11s, YOLO-SDD-s achieves improvements of 0.86%, 1.32%, and 0.93% in AP_50:95_, ${\mathrm{AP}}_{50}$, and ${\mathrm{AP}}_{75}$, respectively, further validating its superior performance in goose detection tasks. These results highlight YOLO-SDD-s’s remarkable ability to accurately detect geese even in challenging environments with occlusions and overlapping instances.

In Table 4, we present a comparison of the performance of various models on the SheepCounter. The YOLO-SDD-s model stands out with an impressive AP_50:95_ score of 62.35%, surpassing all other tested models. This result not only highlights the effectiveness of YOLO-SDD-s in the target detection task but also demonstrates its robustness in counting dense scenes. Additionally, YOLO-SDD-s achieves significant scores in the ${\mathrm{AP}}_{50}$ and ${\mathrm{AP}}_{75}$ metrics, reaching 97.78% and 71.09%, respectively. These results indicate that YOLO-SDD-s demonstrates its strong capability in handling dense small targets and its exceptional generalization in dynamic environments, effectively adapting to varying angles and weather conditions.

**Figure 10:**
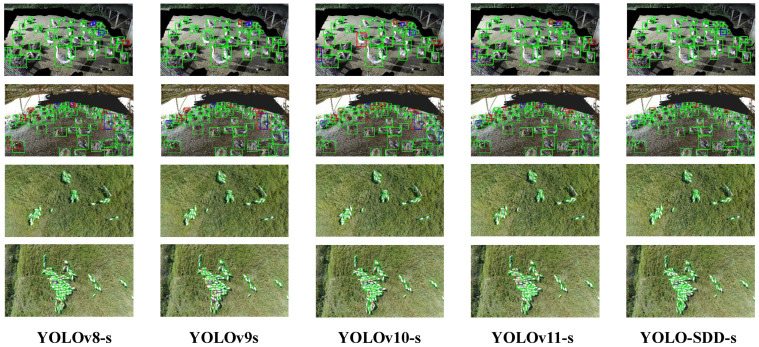
Visualization of detection outcomes for mainstream detectors on GooseDetect and SheepCounter.

The detection results on the two datasets are visualized in Figure 10. On the GooseDetect, the performance differences among models are more pronounced. In scenes with mild occlusion (first row), YOLO-SDD performs best, exhibiting only occasional false positives and misses. YOLOv9 follows as the next best, though with a slightly higher number of false positives. However, YOLOv10 and YOLOv11 show noticeable misses under the same conditions, revealing their limitations in handling occlusion. In the second row, which depicts a more complex environment with a higher number of targets and severe occlusion of some geese, detection difficulty increases significantly. All detectors are affected, displaying varying degrees of false positives and misses. Nevertheless, YOLO-SDD demonstrates greater robustness, effectively reducing both miss and false positive rates, outperforming all compared models. In contrast, YOLOv8’s performance declines most notably in such scenes, with a higher miss rate, indicating its limitations in dense environments and severe occlusion.

On the SheepCounter, YOLO-SDD accurately identifies all targets in low-density scenes (third row), matching YOLOv10’s performance. However, in more crowded scenarios (fourth row), YOLO-SDD again exhibits a clear advantage, achieving the lowest rates of misses and false positives. This superior performance stems from YOLO-SDD’s strong capability in handling dense targets and complex occlusion, particularly due to its accurate and robust feature extraction. Additionally, YOLO-SDD’s design enables it to efficiently address challenges posed by varying angles and weather conditions in dynamic shooting environments, showcasing excellent generalization and practical application potential.

### 3.3. Ablation Study

We conduct ablation experiments on the ChickenFlow dataset to evaluate the impact of each module on network performance. We also analyze the effectiveness of each component, using the unmodified YOLOv8s as the baseline model.

Analyses for WEConv. Table 5 provides a performance comparison of replacing WTConv in WEConv with different convolutional operations, including DWConv and Large Kernel Attention (LKA) [47]. Among these, WTConv achieves the highest AP_50:95_ of 82.93%, surpassing DWConv (82.56%) and LKA (82.59%). Although WTConv has slightly higher FLOPs and Params compared to DWConv, it maintains a balance between computational efficiency and performance, enhancing detection accuracy while keeping computational complexity manageable. DWConv is renowned for its computational efficiency, reflected in the lowest FLOPs (26.8G) and Params (10.3M). However, this efficiency comes at the cost of reduced detection performance. LKA, with a larger receptive field, offers a slight improvement in AP_50:95_. In contrast, WTConv, leverages wavelet transforms to more effectively capture both spatial and frequency domain information, allowing it to handle fine-grained details and contextual information better.

Analyses for LSHead. The performance of the LSHead is evaluated under different middle channel configurations. As shown in Table 6, the model achieves a notable AP_50:95_ of 82.88% with a middle channel size of 64, while maintaining a computational complexity of 22.1G FLOPs and 9.2M parameters. Increasing the middle channel to 128 improves performance of 83.19% with a slightly higher FLOPs of 26.3G and 9.5M parameters. The highest performance is observed at a middle channel set to 256, achieving a peak AP_50:95_ of 83.33%, although at the cost of increased computational demands (42.1G FLOPs). These results indicate that while larger middle channel sizes contribute to improved accuracy, there is a demand for more computational resources. Considering the balance between performance and computational efficiency, we ultimately select the middle channel of 128.

Analyses for OPAM. In analyzing the performance of the OPAM, as shown in Table 7, we evaluate various methods based on AP_50:95_, FLOPs, and the number of parameters. When adding a detection head for the P2 layer without incorporating any attention mechanism, the model’s performance significantly improves to 83.39%, but the computational complexity also increases to 39.7G. Subsequently, we compare different attention mechanisms. The spatial attention results in a slight decrease in AP_50:95_ to 83.19%, with FLOPs at 31.4G and parameter count at 11.5M. Channel attention further enhances performance to 83.31%, while FLOPs remain at 31.5G. Utilizing CBAM increases the AP_50:95_ to 83.42%, while keeping the FLOPs and parameters similar. Ultimately, OPAM achieves the highest performance with an AP_50:95_ of 83.52%, FLOPs at 33.1G, and parameters at 11.4M after introducing the occlusion aware mechanism. This underscores OPAM’s significant advantage in enhancing detection accuracy, particularly in challenging occluded and dense scenes, thus validating its effectiveness.

Ablation study for each component. In the ablation study of each component, as shown in Table 8, the baseline achieves an AP_50:95_ of 82.14%. After introducing the occlusion loss (Row 2), the model’s performance slightly improves to 82.49%. When WEConv is added (Row 3), the performance significantly increases to 83.44%, with FLOPs reduced to 26.8G, demonstrating its advantage in capturing fine-grained information. The introduction of OPAM (Row 4) further enhances the performance to 83.64%, despite an increase in computational complexity to 33.1 G. Incorporating LSHead (Row 5) yields an AP_50:95_ of 83.38% with reduced FLOPs (26.3G) and parameters (9.5M). By combining the occlusion loss, WEConv, and OPAM (Row 6), the model achieves an AP_50:95_ of 83.73%, indicating that these components work synergistically to effectively enhance detection performance. Ultimately, when all components are used together (Row 9), the model reaches the highest AP_50:95_ value of 84.27% while maintaining a reasonable FLOPs (28.8G) and parameter count (9M). This series of experimental results demonstrates that the effective combination of these components can significantly improve model performance.

### 3.4. Activation Maps of Different Attentions

**Figure 11:**
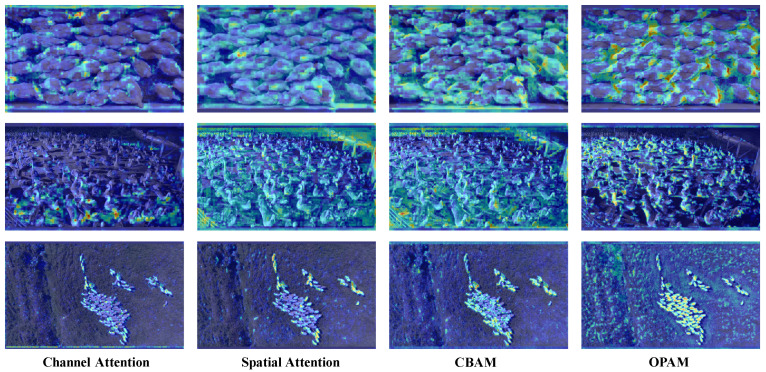
Visualizations of feature maps generated with different attention mechanisms using Grad-CAM++ [48].

In Figure 11, we present the activation maps generated by models employing four different attention mechanisms: channel attention, spatial attention, CBAM, and our proposed OPAM. From the visualizations, it is clear that OPAM outperforms the other methods in emphasizing dense and occluded regions. Channel attention focuses on fewer targets, which may lead to missed detections. Spatial attention, on the other hand, emphasizes target localization with a broader attention scope, but lacks refined feature extraction capabilities. CBAM combines the advantages of both channel and spatial attention, achieving better performance in target localization and feature refinement. However, it still falls short in handling dense scenes and severe occlusions, as it fails to effectively highlight features in occluded areas. In contrast, OPAM exhibits exceptional capability in capturing densely occluded regions, enabling the model to precisely focus on challenging areas prone to occlusion. This improves the localization and recognition performance in complex and crowded environments.

## 4. Conclusions

In this paper, we propose YOLO-SDD, a network specifically designed for single-class dense object detection with occlusion awareness. The model incorporates three key innovations: the WEConv for improved feature extraction under dense occlusion, the OPAM to enhance feature discrimination in complex occlusion scenarios, and the LS Head optimized for single-class detection tasks. Extensive experiments on the ChickenFlow, GooseDetect, and SheepCounter datasets demonstrated that YOLO-SDD outperforms the state-of-the-art detectors, especially in challenging, densely occluded environments. The ablation studies further validate the contribution of each module to the overall performance.

Our future work will focus on extending the model’s applicability to different livestock scenarios and species to enhance its practical utility. Although YOLO-SDD reduces computational complexity compared to the baseline YOLOv8, deployment on resource-constrained devices remains challenging. Techniques such as model pruning and knowledge distillation could be explored to further alleviate computational burden. Additionally, integrating YOLO-SDD with multi-object tracking technologies could improve real-time monitoring accuracy and stability in dynamic scenes.   

## Figures and Tables

**Table 1:** Performance comparison of mainstream real-time object detectors on ChickenFlow.

Model	AP_50:95_ (%)	AP_50_ (%)	AP_75_ (%)	FLOPs	Params (M)
YOLOv5n	75.27	95.86	88.09	4.1 G	1.9
YOLOv5s	78.64	96.12	90.48	16.5 G	7.2
YOLOv5m	81.41	96.38	91.29	49.0 G	21.2
YOLOv7-tiny	74.17	95.54	86.77	13.7 G	6.2
YOLOv7	79.07	96.48	89.33	104.7 G	36.9
YOLOv8n	79.01	96.28	90.90	8.7 G	3.2
YOLOv8s	82.14	96.85	92.02	28.6 G	11.2
YOLOv8m	84.03	96.44	91.95	78.9 G	25.9
YOLOv9t	77.58	96.20	89.25	7.7 G	2.0
YOLOv9s	81.04	96.65	91.55	26.7 G	7.2
YOLOv9m	83.51	96.76	92.61	76.8 G	20.1
YOLOv10n	79.97	96.66	91.51	8.4 G	2.7
YOLOv10s	82.64	96.45	92.57	24.8 G	8.6
YOLOv10m	84.55	96.84	92.73	64.0 G	16.5
Mamba-YOLO-T	79.33	96.07	90.59	14.3 G	6.1
Mamba-YOLO-B	82.43	96.76	92.02	49.7 G	21.8
YOLOv11n	79.50	96.81	90.96	6.5 G	2.6
YOLOv11s	82.40	97.01	92.00	21.5 G	9.4
YOLOv11m	84.01	96.86	92.55	68.0 G	20.1
YOLO-SDD-n	81.19	96.90	92.01	11.4 G	2.6
YOLO-SDD-s	84.27	97.17	93.24	28.8 G	9.0
YOLO-SDD-m	85.65	97.30	93.06	77.1 G	22.0

**Table 2:** Comparison of experimental results on dense occlusion scenes (OR > 0.5 and objects per image > 40) from the ChickenFlow dataset. The bold values in the table represent the best results for each metric.

Model	AP_50:95_ (%)	AP_50_ (%)	AP_75_ (%)
YOLOv5s	71.43	93.06	83.12
YOLOv8s	72.47	93.17	83.77
YOLOv9s	72.92	93.28	82.66
YOLOv10s	73.52	93.53	83.97
Mamba-YOLO-T	70.29	92.70	81.15
YOLOv11s	72.54	93.26	82.57
YOLO-SDD-s	**75.60**	**94.04**	**85.38**

**Table 3:** Comparison of results on GooseDetect. The bold values in the table represent the best results for each metric.

Model	AP_50:95_ (%)	AP_50_ (%)	AP_75_ (%)
YOLOv5s	53.26	90.01	55.97
YOLOv8s	54.85	90.45	58.01
YOLOv9s	56.22	91.27	60.36
YOLOv10s	54.96	90.67	58.81
Mamba-YOLO-T	53.76	89.77	56.59
YOLOv11s	55.61	90.39	59.75
YOLO-SDD-s	**56.47**	**91.71**	**60.68**

**Table 4:** Comparison of results on SheepCounter. The bold values in the table represent the best results for each metric.

Model	AP_50:95_ (%)	AP_50_ (%)	AP_75_ (%)
YOLOv5s	58.87	95.62	64.84
YOLOv8s	60.72	97.38	68.21
YOLOv9s	61.97	97.19	70.95
YOLOv10s	61.45	97.57	69.81
Mamba-YOLO-T	59.58	96.93	67.80
YOLOv11s	60.68	97.26	69.28
YOLO-SDD-s	**62.35**	**97.78**	**71.09**

**Table 5:** Analyses for WEConv. The bold values in the table represent the best results for each metric.

Method	AP_50:95_ (%)	FLOPs	Params
DWConv	82.56	**26.8 G**	**10.3 M**
LKA	82.59	27.4 G	10.4 M
WTConv	**82.93**	27 G	10.4 M

**Table 6:** Analyses for LSHead. The bold values in the table represent the best results for each metric.

Method	AP_50:95_ (%)	FLOPs	Params
middle channel = 64	82.88	**22.1 G**	**9.2 M**
middle channel = 128	83.19	26.3 G	9.5 M
middle channel = 256	**83.33**	42.1 G	10.5 M

**Table 7:** Analyses for OPAM. The bold values in the table represent the best results for each metric.

Method	AP_50:95_ (%)	FLOPs	Params
Baseline	82.14	**28.6 G**	11.2 M
P2	83.39	39.7 G	**10.8 M**
Spatial Attention	83.19	31.4 G	11.4 M
Channel Attention	83.31	31.5 G	11.4 M
CBAM	83.42	31.5 G	11.4 M
OPAM	**83.52**	33.1 G	11.4 M

**Table 8:** Ablation study of each component. The bold values in the table represent the best results for each metric.

#	Occlusion Loss	WEConv	OPAM	LS Head	AP_50:95_ (%)	FLOPs	Params
1					82.14	28.6 G	11.2 M
2	✓				82.49	28.6 G	11.2 M
3	✓	✓			83.44	26.8 G	10.4 M
4	✓		✓		83.64	33.1 G	11.4 M
5	✓			✓	83.38	26.3 G	9.5 M
6	✓	✓	✓		83.73	31.4 G	10.7 M
7	✓	✓		✓	83.66	**24.5 G**	**8.8 M**
8	✓		✓	✓	83.68	30.4 G	9.8 M
9	✓	✓	✓	✓	**84.27**	28.8 G	9.0 M

## Data Availability

The ChickenFlow datasets presented in this study can be accessed at the following link: [https://drive.google.com/drive/folders/1Zlwdwq6zLf3U_MOz4jR0BW8PyrUfKuiK?usp=sharing] (accessed on 17 April 2025). The GooseDetect dataset is publicly available at https://doi.org/10.57760/sciencedb.14382. The SheepCounter dataset can be accessed at https://universe.roboflow.com/riisprivate/sheepcounter/dataset/11.

## Appendix A

Table A1 summarizes the YOLO-SDD’s architecture, focusing on key layers and their configurations that are critical for replication. Module-specific arguments are formatted as follows: WTConv/Conv: [output channels, kernel size, stride]; C2f: [output channels, shortcut]; SPPF: [output channels, pooling size]; Upsample: [scale factor, mode]; Concat: [dimension]; LS Head: [classes, channels].

**Table A1:** YOLO-SDD model configuration.

Layer	Repeats	Module	Arguments
1	1	WTConv	[64, 3, 2]
2	1	WTConv	[128, 3, 2]
3	3	C2f	[128, True]
4	1	WTConv	[256, 3, 2]
5	6	C2f	[256, True]
6	1	WTConv	[512, 3, 2]
7	6	C2f	[512, True]
8	1	WTConv	[1024, 3, 2]
9	3	C2f	[1024, True]
10	1	SPPF	[1024, 5]
11	1	Upsample	[2, “nearest”]
12	1	Concat	[11, 7]
13	3	C2f	[512]
14	1	Upsample	[2, “nearest”]
15	1	Concat	[14, 5]
16	3	C2f	[256]
17	1	Upsample	[2, “nearest”]
18	1	OPAM	[17, 2]
19	1	Conv	[128, 3, 2]
20	1	Concat	[19, 16]
21	3	C2f	[256]
22	1	Conv	[256, 3, 2]
23	1	Concat	[22, 13]
24	3	C2f	[512]
25	1	Conv	[512, 3, 2]
26	1	Concat	[25, 10]
27	3	C2f	[1024]
28	1	LSHead	[80, 128]

## References

[1] Fernandes A.F.A., Dórea J.R.R., Rosa G.J.d.M. (2020). Image analysis and computer vision applications in animal sciences: An overview. Front. Vet. Sci..

[2] Okinda C., Nyalala I., Korohou T., Okinda C., Wang J., Achieng T., Wamalwa P., Mang T., Shen M. (2020). A review on computer vision systems in monitoring of poultry: A welfare perspective. Artif. Intell. Agric..

[3] Constance D.H., Martinez-Gomez F., Aboites-Manrique G., Bonanno A. (2013). The problems with poultry production and processing. The Ethics and Economics of Agrifood Competition.

[4] Racewicz P., Ludwiczak A., Skrzypczak E., Składanowska-Baryza J., Biesiada H., Nowak T., Nowaczewski S., Zaborowicz M., Stanisz M., Ślósarz P. (2021). Welfare Health and Productivity in Commercial Pig Herds. Animals.

[5] Tassinari P., Bovo M., Benni S., Franzoni S., Poggi M., Mammi L.M.E., Mattoccia S., Di Stefano L., Bonora F., Barbaresi A. (2021). A computer vision approach based on deep learning for the detection of dairy cows in free stall barn. Comput. Electron. Agric..

[6] Sundaram N., Meena S.D. (2023). Integrated animal monitoring system with animal detection and classification capabilities: A review on image modality, techniques, applications, and challenges. Artif. Intell. Rev..

[7] Yin C., Tan X., Li X., Cai M., Chen W. (2025). Only Detect Broilers Once (ODBO): A Method for Monitoring and Tracking Individual Behavior of Cage-Free Broilers. Agriculture.

[8] Liu Y., Li W., Liu X., Li Z., Yue J. (2024). Deep learning in multiple animal tracking: A survey. Comput. Electron. Agric..

[9] Yang X., Bist R.B., Paneru B., Liu T., Applegate T., Ritz C., Kim W., Regmi P., Chai L. (2024). Computer vision-based cybernetics systems for promoting modern poultry farming: A critical review. Comput. Electron. Agric..

[10] Dalal N., Triggs B. Histograms of oriented gradients for human detection. Proceedings of the 2005 IEEE Computer Society Conference on Computer Vision and Pattern Recognition (CVPR’05).

[11] Zou X. A review of object detection techniques. Proceedings of the 2019 International Conference on Smart Grid and Electrical Automation (ICSGEA).

[12] Ren S., He K., Girshick R., Sun J. (2016). Faster R-CNN: Towards real-time object detection with region proposal networks. IEEE Trans. Pattern Anal. Mach. Intell..

[13] Liu W., Anguelov D., Erhan D., Szegedy C., Reed S., Fu C.Y., Berg A.C. (2016). Ssd: Single shot multibox detector. Proceedings of the Computer Vision–ECCV 2016: 14th European Conference.

[14] Redmon J., Divvala S., Girshick R., Farhadi A. You only look once: Unified, real-time object detection. Proceedings of the IEEE Conference on Computer Vision and Pattern Recognition.

[15] Tu S., Yuan W., Liang Y., Wang F., Wan H. (2021). Automatic detection and segmentation for group-housed pigs based on PigMS R-CNN. Sensors.

[16] Song S., Liu T., Wang H., Hasi B., Yuan C., Gao F., Shi H. (2022). Using pruning-based YOLOv3 deep learning algorithm for accurate detection of sheep face. Animals.

[17] Yu R., Wei X., Liu Y., Yang F., Shen W., Gu Z. (2024). Research on automatic recognition of dairy cow daily behaviors based on deep learning. Animals.

[18] Cao L., Xiao Z., Liao X., Yao Y., Wu K., Mu J., Li J., Pu H. (2021). Automated chicken counting in surveillance camera environments based on the point supervision algorithm: LC-DenseFCN. Agriculture.

[19] Lai J., Liang Y., Kuang Y., Xie Z., He H., Zhuo Y., Huang Z., Zhu S., Huang Z. (2023). IO-YOLOv5: Improved pig detection under various illuminations and heavy occlusion. Agriculture.

[20] Bodla N., Singh B., Chellappa R., Davis L.S. Soft-NMS–improving object detection with one line of code. Proceedings of the IEEE International Conference on Computer Vision.

[21] Hao W., Zhang L., Han M., Zhang K., Li F., Yang G., Liu Z. (2023). YOLOv5-SA-FC: A novel pig detection and counting method based on shuffle attention and focal complete intersection over union. Animals.

[22] Yang J., Zhang T., Fang C., Zheng H., Ma C., Wu Z. (2024). A detection method for dead caged hens based on improved YOLOv7. Comput. Electron. Agric..

[23] Sun D., Zhang L., Wang J., Liu X., Wang Z., Hui Z., Wang J. (2024). Efficient and accurate detection of herd pigs based on Ghost-YOLOv7-SIoU. Neural Comput. Appl..

[24] Chen Z., Hou Y., Yang C. Research on Identification of Sick Chicken Based on Multi Region Deep Features Fusion. Proceedings of the 2021 6th International Conference on Computational Intelligence and Applications (ICCIA).

[25] Wan Z., Tian F., Zhang C. (2023). Sheep face recognition model based on deep learning and bilinear feature fusion. Animals.

[26] Zhao S., Bai Z., Huo L., Han G., Duan E., Gong D., Gao L. (2024). Automatic Perception of Typical Abnormal Situations in Cage-Reared Ducks Using Computer Vision. Animals.

[27] Jie D., Wang J., Wang H., Lv H., He J., Wei X. (2025). Real-time recognition research for an automated egg-picking robot in free-range duck sheds. J. Real-Time Image Process..

[28] Gao Y., Yan K., Dai B., Sun H., Yin Y., Liu R., Shen W. (2023). Recognition of aggressive behavior of group-housed pigs based on CNN-GRU hybrid model with spatio-temporal attention mechanism. Comput. Electron. Agric..

[29] Shang C., Wu F., Wang M., Gao Q. (2022). Cattle behavior recognition based on feature fusion under a dual attention mechanism. J. Vis. Commun. Image Represent..

[30] Geng H., Hou Z., Liang J., Li X., Zhou X., Xu A. (2024). Motion focus global–local network: Combining attention mechanism with micro action features for cow behavior recognition. Comput. Electron. Agric..

[31] Feng Y., Li W., Guo Y., Wang Y., Tang S., Yuan Y., Shen L. (2024). GooseDetect lion: A Fully Annotated Dataset for Lion-head Goose Detection in Smart Farms. Sci. Data.

[32] Doll O., Loos A. Comparison of Object Detection Algorithms for Livestock Monitoring of Sheep in UAV images. Proceedings of the Camera Traps, AI, and Ecology—3rd International Workshop.

[33] Sekachev B., Zhavoronkov A., Manovich N. (2019). Computer vision annotation tool: A universal approach to data annotation. Intel [Internet].

[34] Jocher G., Chaurasia A., Qiu J. Ultralytics YOLO, 2023. Original-Date: 2022-09-11T16:39:45Z. https://github.com/ultralytics/ultralytics.

[35] Wang X., Xiao T., Jiang Y., Shao S., Sun J., Shen C. Repulsion Loss: Detecting Pedestrians in a Crowd. Proceedings of the 2018 IEEE/CVF Conference on Computer Vision and Pattern Recognition.

[36] Han K., Wang Y., Tian Q., Guo J., Xu C., Xu C. Ghostnet: More features from cheap operations. Proceedings of the IEEE/CVF Conference on Computer Vision and Pattern Recognition.

[37] Chollet F. Xception: Deep learning with depthwise separable convolutions. Proceedings of the IEEE Conference on Computer Vision and Pattern Recognition.

[38] Finder S.E., Amoyal R., Treister E., Freifeld O., Leonardis A., Ricci E., Roth S., Russakovsky O., Sattler T., Varol G. (2025). Wavelet Convolutions for Large Receptive Fields. Proceedings of the Computer Vision–ECCV 2024, Milan, Italy, 29 September–4 October 2024.

[39] Lin Y., Liu M., Yang C., Li S., Zhang W. (2023). AC-YOLO: A Safety Helmet Detection based on YOLOX. Proceedings of the 2022 4th International Conference on Robotics, Intelligent Control and Artificial Intelligence.

[40] Tian Z., Shen C., Chen H., He T. FCOS: Fully Convolutional One-Stage Object Detection. Proceedings of the 2019 IEEE/CVF International Conference on Computer Vision (ICCV).

[41] Jocher G., Stoken A., Borovec J., Changyu L., Hogan A., Diaconu L., Poznanski J., Yu L., Rai P., Ferriday R. (2020). ultralytics/yolov5: v3. 0. Zenodo.

[42] Wang C.Y., Bochkovskiy A., Liao H.Y.M. YOLOv7: Trainable Bag-of-Freebies Sets New State-of-the-Art for Real-Time Object Detectors. Proceedings of the 2023 IEEE/CVF Conference on Computer Vision and Pattern Recognition (CVPR).

[43] Wang C.Y., Yeh I.H., Mark Liao H.Y., Leonardis A., Ricci E., Roth S., Russakovsky O., Sattler T., Varol G. (2024). YOLOv9: Learning What You Want to Learn Using Programmable Gradient Information. Proceedings of the Computer Vision–ECCV 2024, Milan, Italy, 29 September–4 October 2024.

[44] Wang A., Chen H., Liu L., Chen K., Lin Z., Han J., Ding G. (2024). YOLOv10: Real-Time End-to-End Object Detection. arXiv.

[45] Khanam R., Hussain M. (2024). Yolov11: An overview of the key architectural enhancements. arXiv.

[46] Wang Z., Li C., Xu H., Zhu X. (2024). Mamba YOLO: SSMs-based YOLO for object detection. arXiv.

[47] Guo M.H., Lu C.Z., Liu Z.N., Cheng M.M., Hu S.M. (2023). Visual attention network. Comput. Vis. Media.

[48] Chattopadhay A., Sarkar A., Howlader P., Balasubramanian V.N. Grad-cam++: Generalized gradient-based visual explanations for deep convolutional networks. Proceedings of the 2018 IEEE Winter Conference on Applications of Computer Vision (WACV).

